# The complete genome sequence of *Eubacterium limosum* SA11, a metabolically versatile rumen acetogen

**DOI:** 10.1186/s40793-016-0147-9

**Published:** 2016-03-15

**Authors:** William J. Kelly, Gemma Henderson, Diana M. Pacheco, Dong Li, Kerri Reilly, Graham E. Naylor, Peter H. Janssen, Graeme T. Attwood, Eric Altermann, Sinead C. Leahy

**Affiliations:** Rumen Microbiology, Animal Science, AgResearch Limited, Grasslands Research Centre, Tennent Drive, Private Bag 11008, Palmerston North, 4442 New Zealand

**Keywords:** Acetogen, Methane mitigation, Rumen, *Eubacterium limosum*, Wood–Ljungdahl pathway, Butyrate

## Abstract

Acetogens are a specialized group of anaerobic bacteria able to produce acetate from CO_2_ and H_2_ via the Wood–Ljungdahl pathway. In some gut environments acetogens can compete with methanogens for H_2_, and as a result rumen acetogens are of interest in the development of microbial approaches for methane mitigation. The acetogen *Eubacterium limosum* SA11 was isolated from the rumen of a New Zealand sheep and its genome has been sequenced to examine its potential application in methane mitigation strategies, particularly in situations where hydrogenotrophic methanogens are inhibited resulting in increased H_2_ levels in the rumen. The 4.15 Mb chromosome of SA11 has an average G + C content of 47 %, and encodes 3805 protein-coding genes. There is a single prophage inserted in the chromosome, and several other gene clusters appear to have been acquired by horizontal transfer. These include genes for cell wall glycopolymers, a type VII secretion system, cell surface proteins and chemotaxis. SA11 is able to use a variety of organic substrates in addition to H_2_/CO_2_, with acetate and butyrate as the principal fermentation end-products, and genes involved in these metabolic pathways have been identified. An unusual feature is the presence of 39 genes encoding trimethylamine methyltransferase family proteins, more than any other bacterial genome. Overall, SA11 is a metabolically versatile organism, but its ability to grow on such a wide range of substrates suggests it may not be a suitable candidate to take the place of hydrogen-utilizing methanogens in the rumen.

**Electronic supplementary material:**

The online version of this article (doi:10.1186/s40793-016-0147-9) contains supplementary material, which is available to authorized users.

## Introduction

Methane produced by methanogenic archaea during the fermentation of plant material in the rumen is widely regarded as a significant contributor to anthropogenic greenhouse gas emissions from ruminant livestock. Several approaches to reduce CH_4_ emissions from farmed animals are currently being investigated, and the genomes of several rumen methanogens have been sequenced to support strategies designed to reduce the number or metabolic activity of methanogens in the rumen [1]. Hydrogen is necessary for methanogenesis and this has led to proposals that organisms which compete with methanogens for H_2_ could be used to reduce CH_4_ production [1–4]. Anaerobic bacteria capable of reductive acetogenesis are of particular interest as these organisms use the Wood–Ljungdahl pathway to synthesize acetyl-CoA by the reduction of CO or CO_2_ and H_2_ with the resulting acetate available to the animal [5]. Thus an additional strategy proposed is the use of acetogens in conjunction with methanogen inhibition so that hydrogen does not accumulate and inhibit fermentation.

In some gut environments acetogens can compete with methanogens for H_2_, although the process is not energetically favoured by conditions found in the mature rumen [6]. Nevertheless, reductive acetogenesis has been shown to occur in batch cultures when methanogenesis is inhibited and acetogens are added [7, 8]. Acetogenic bacteria are thought to be the dominant hydrogenotrophs in early rumen microbiota [9, 10], and understanding their ecology in the developing digestive tract of ruminants may reveal key features that lead to the prevalence of methanogens and the restriction of homoacetogens in the adult rumen. Consequently, rumen acetogens are of interest in the development of microbial approaches to methane mitigation. Several acetogens have been isolated from the rumen [2], and analyses of sequences of formyltetrahydrofolate synthetase, a key enzyme of the Wood–Ljungdahl pathway, indicate that additional species remain uncultured [11, 12]. Here we present the genome sequence of *E. limosum* strain SA11 isolated from the rumen of a sheep [2].

## Organism information

### Classification and features

**Fig. 1:**
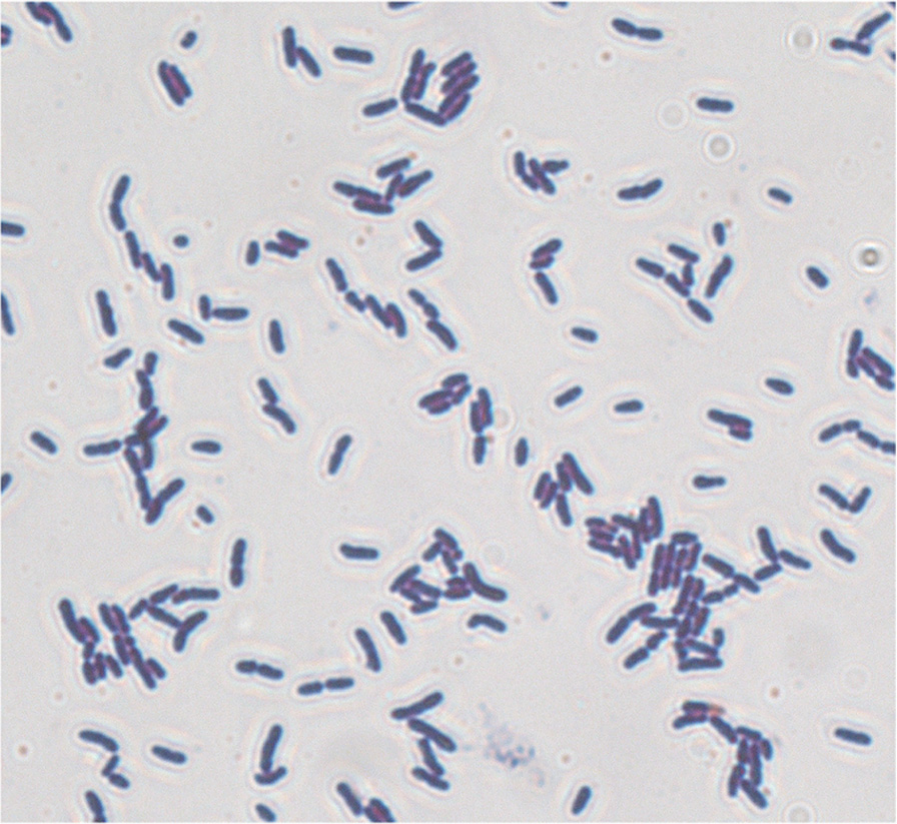
Morphology of *E. limosum* SA11. Micrograph of *E. limosum* SA11 cells captured at 100x magnification

**Fig. 2:**
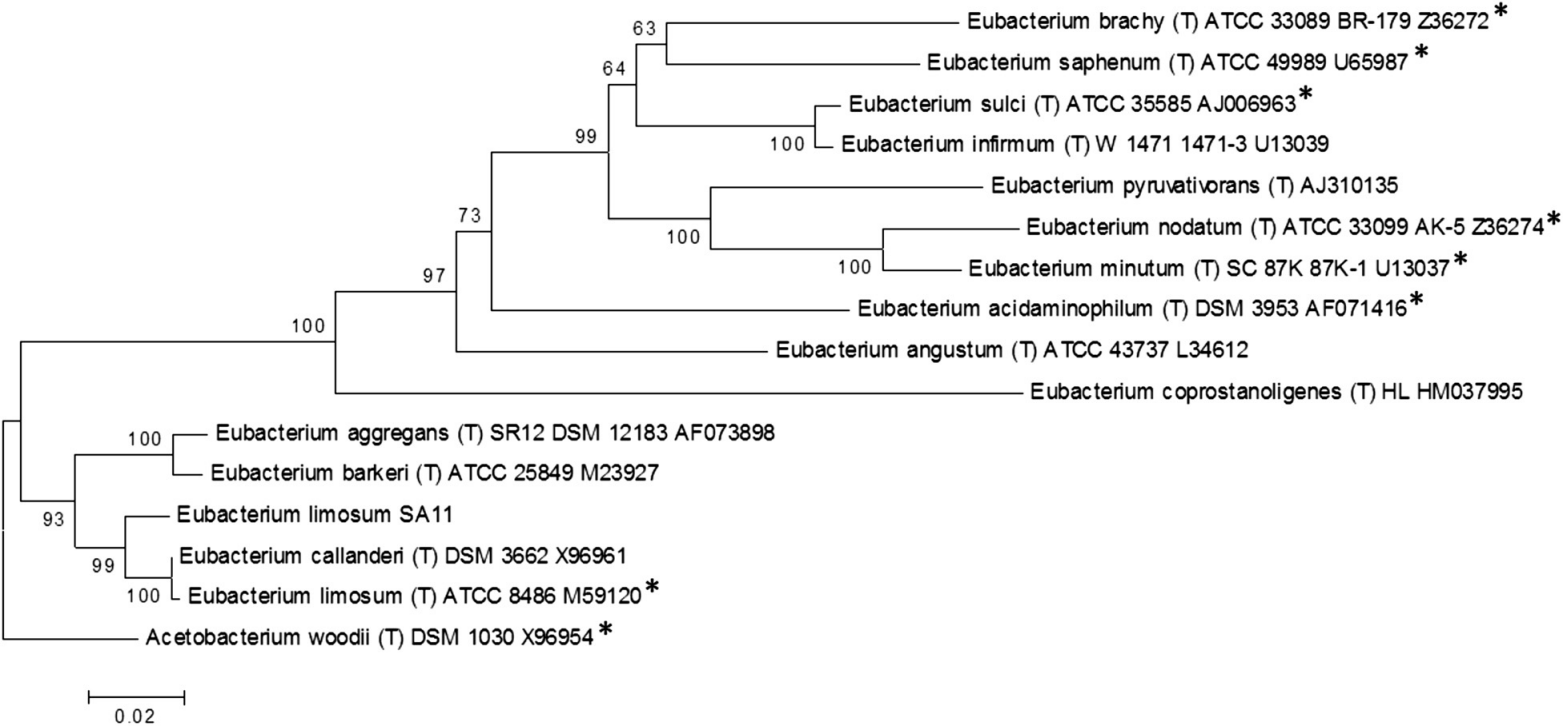
Phylogenetic tree highlighting the position of *E. limosum* SA11 relative to the type strains of the other *Eubacterium* species. The evolutionary history was inferred using the Neighbor-Joining method [43]. The optimal tree with the sum of branch length = 0.83983608 is shown. The percentage of replicate trees in which the associated taxa clustered together in the bootstrap test (1000 replicates) is shown next to the branches [44]. The tree is drawn to scale, with branch lengths in the same units as those of the evolutionary distances used to infer the phylogenetic tree. The evolutionary distances were computed using the Kimura 2-parameter method [45] and are in the units of the number of base substitutions per site. The rate variation among sites was modeled with a gamma distribution (shape parameter = 1). The analysis involved 16 nucleotide sequences. All positions containing gaps and missing data were eliminated. There were a total of 1214 positions in the final dataset. Evolutionary analyses were conducted in MEGA6 [46]. Species with strain genome sequencing projects registered in the Genomes Online Database (GOLD) [47] are labeled with an asterisk

*Eubacterium limosum* SA11 was isolated from the rumen of a New Zealand sheep grazing fresh forage [2], and was originally described as sheep acetogen SA11 but not characterized further. Cells of SA11 are Gram positive non-motile rods occurring singly and in pairs (Fig. 1). The 16S rRNA from SA11 is 97 % similar to the *E. limosum* type strain ATCC 8486^T^ which was isolated from human faeces, and as such SA11 can be considered as a rumen strain of *E. limosum* (Fig. 2). Strains of *E. limosum* have been isolated from various anaerobic environments including the gastrointestinal tract of various animals, sewage and mud [13, 14]. *E. limosum* was the first rumen acetogen to be isolated [13], and this strain (RF) was characterized [15, 16] and used in co-culture studies with the pectin-degrading rumen bacterium *Lachnospira multipara* [17]. These studies showed *E. limosum* to be a metabolically versatile bacterium able to grow on a wide variety of compounds including CO, CO_2_/H_2_, hexoses, pentoses, alcohols, methyl-containing compounds, formate, lactate, and some amino acids. Acetate and butyrate are the main fermentation end-products, although butyrate production is low when grown on CO_2_/H_2_ [13]. Additional characteristics of strain SA11 are shown in Table 1.

**Table 1:** Classification and general features of *Eubacterium limosum* SA11 [48]

MIGS ID	Property	Term	Evidence code^a^
	Classification	Domain: Bacteria	TAS [49]
		Phylum: *Firmicutes*	TAS [50, 51]
		Class: *Clostridia*	TAS [52, 53]
		Order: *Clostridiales*	TAS [54, 55]
		Family: *Eubacteriaceae*	TAS [53, 56]
		Genus: *Eubacterium*	TAS [14, 54, 57]
		Species: *limosum*	TAS [58]
		strain: SA11	
	Gram stain	Positive	TAS [14]
	Cell shape	Rod	TAS [14]
	Motility	Non-motile	TAS [14]
	Sporulation	Not reported	NAS
	Temperature range	30-45 °C	NAS
	Optimum temperature	37 °C	NAS
	pH range; Optimum	5.0-7.5; 7.0	NAS
	Carbon source	Glucose, fructose, lactate, methanol, 3,4,5 trimethoxybenzoic acid	IDA
MIGS-6	Habitat	Sheep rumen	TAS [2]
MIGS-6.3	Salinity	Not reported	
MIGS-22	Oxygen requirement	Anaerobic	IDA
MIGS-15	Biotic relationship	Symbiont	TAS [2]
MIGS-14	Pathogenicity	Non-pathogen	NAS
MIGS-4	Geographic location	Palmerston North, New Zealand	IDA
MIGS-5	Sample collection	Not reported	
MIGS-4.1	Latitude	-40.35 (40°21'00"S)	IDA
MIGS-4.2	Longitude	+175.61 (175°36'36"E)	IDA
MIGS-4.4	Altitude	30 M	IDA

^a^Evidence codes - *IDA* Inferred from Direct Assay, *TAS* Traceable Author Statement (i.e., a direct report exists in the literature), *NAS* Non-traceable Author Statement (i.e., not directly observed for the living, isolated sample, but based on a generally accepted property for the species, or anecdotal evidence). These evidence codes are from the Gene Ontology project [59]

## Genome sequencing information

### Genome project history

*Eubacterium limosum* SA11 was selected for genome sequencing as an example of a rumen acetogen isolated in New Zealand with potential application in methane mitigation strategies. A summary of the genome project information is shown in Table 2 and Additional file 1: Table S1 .

**Table 2:** Project information

MIGS ID	Property	Term
MIGS-31	Finishing quality	High-quality, closed genome
MIGS-28	Libraries used	Paired-end library
MIGS-29	Sequencing platforms	454 GS FLX Titanium chemistry
MIGS-31.2	Fold coverage	43×
MIGS-30	Assemblers	Newbler
MIGS-32	Gene calling method	Glimmer and BLASTX
	Locus Tag	ACH52_
	Genbank ID	CP011914
	Genbank Date of Release	23^rd^ December 2015
	GOLD ID	Gp0125209
	BIOPROJECT	PRJNA280903
MIGS 13	Source Material Identifier	*Eubacterium limosum* SA11
	Project relevance	Ruminant methane emissions

### Growth conditions and genomic DNA preparation

**Fig. 3:**
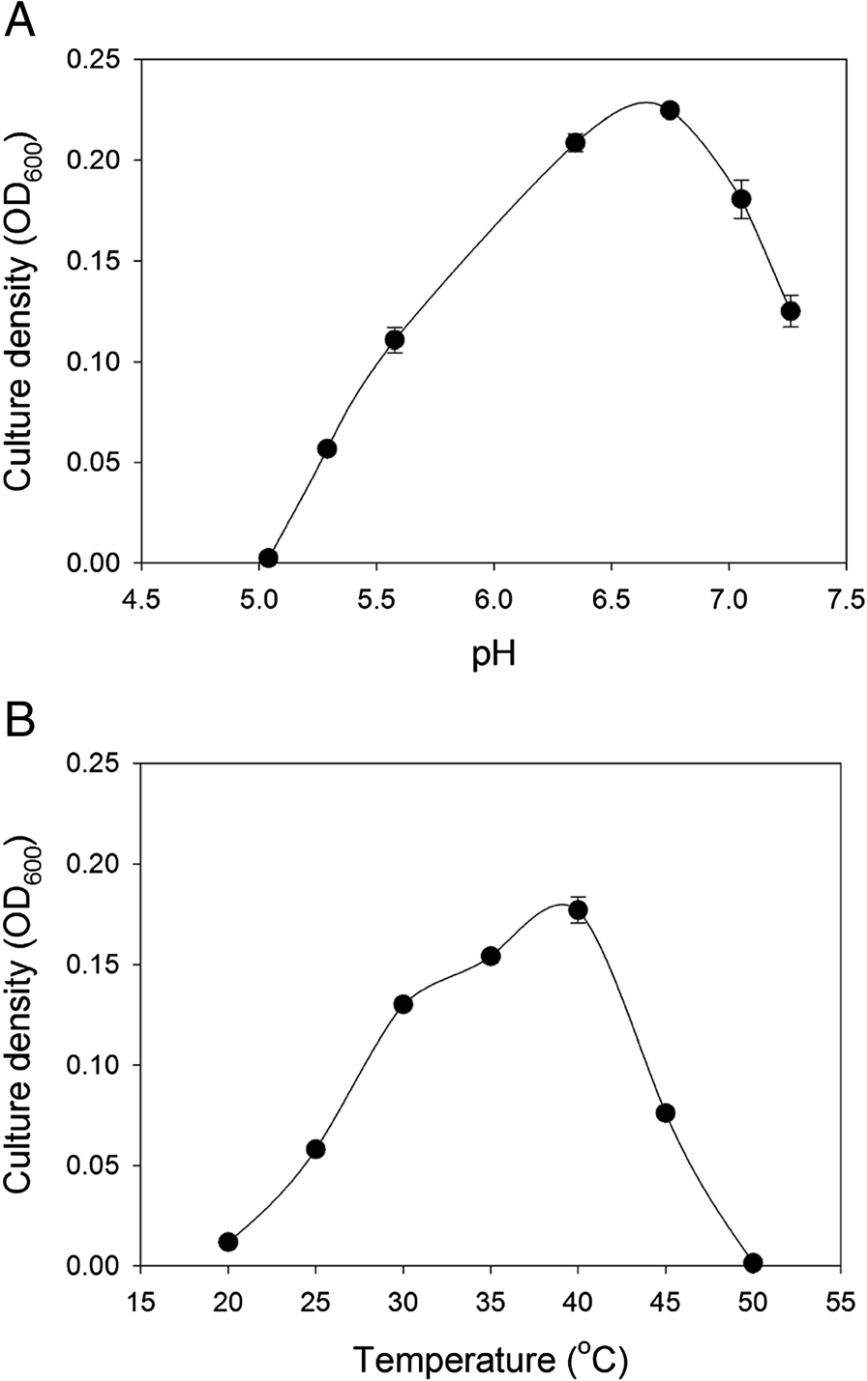
**a** Culture density achieved in 40 h by SA11 growing with hydrogen in media with different pH values. Points indicate means of three replicates, with one standard error on either side of the mean. **b** Culture density achieved in 40 h by SA11 growing with hydrogen at different temperatures. Points indicate means of three replicates, with one standard error on either side of the mean

Strain SA11 was able to grow in CO_2_-containing media with the following energy sources (all tested at 10 mM): hydrogen, formate, D-glucose, D-fructose, D-xylose, D-ribose, maltose, pyruvate, L-lactate, methanol, vanillate, syringate, and 3,4,5-trimethoxybenzoate. Growth was assessed as an increase in culture density compared to cultures that contained none of the added energy sources. The following did not support growth: D-mannose, D-galactose, L-arabinose, L-rhamnose, D-cellobiose, sucrose, lactose, melibiose, raffinose, D-mannitol, D-sorbitol, glycerol, succinate, ethanol, ethylene glycol, 2-methoxyethanol, gallate, ferulate, aesculin, glycine, L-glutamate, and betaine. Glucose and methanol are the best substrates and support the growth of SA11 to a high cell density. Strain SA11 grew most rapidly at pH values of 6.5 to 7.0 (Fig. 3a) and at a temperature of about 40 °C (Fig. 3b). These are typical of its rumen environment.

**Fig. 4:**
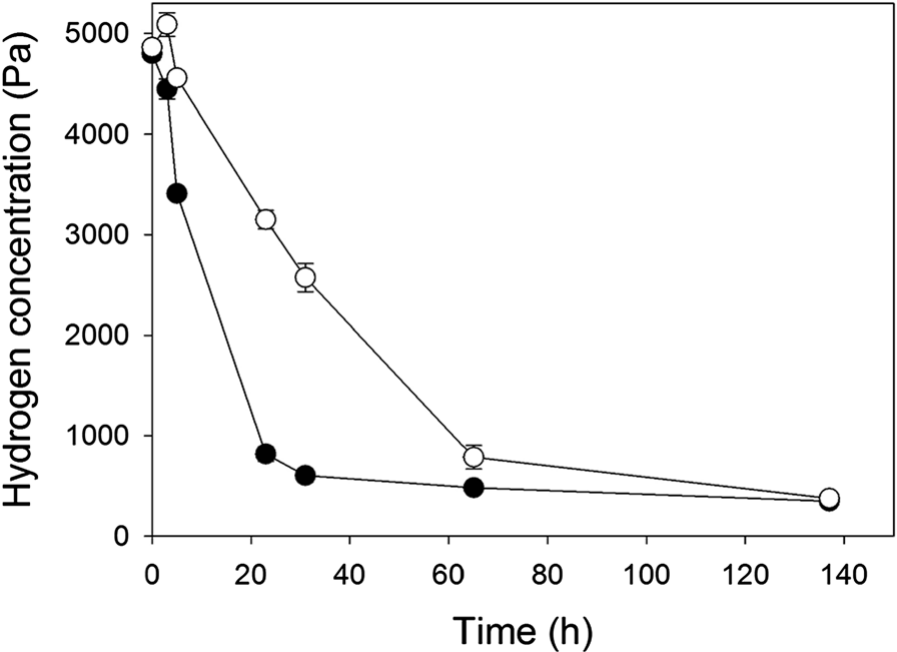
Use of hydrogen by suspensions of hydrogen-grown (○) or glucose-grown (●) cells of SA11. Points indicate means of five replicates, with one standard error on either side of the mean

Cells of SA11 grown with hydrogen or glucose were resuspended in fresh medium and 5000 Pa hydrogen was added to the culture headspace. Cells grown with both substrates were able to used gaseous hydrogen to a threshold concentration of 347 to 375 Pa (Fig. 4), at which point hydrogen use stopped. These concentrations are equivalent to 2.10 to 2.25 μM dissolved hydrogen. Normal ruminal hydrogen concentrations can exceed this directly after feeding, but are also below this over the animal feeding cycle [18], meaning that strain SA11 probably can grow as a hydrogen-dependent homoacetogen at times when hydrogen concentrations are high in the rumen.

SA11 cells for genome sequencing were grown in RM02 medium [19] with 10 mM glucose and 0.1 % yeast extract but without rumen fluid. Culture purity was confirmed by Gram stain and sequencing of the 16S rRNA gene. Genomic DNA was extracted from freshly grown cells by standard cell lysis methods using lysozyme, proteinase K and sodium dodecyl sulphate, followed by phenol-chloroform extraction, and purified using the Qiagen Genomic-Tip 500 Maxi kit (Qiagen, Hilden, Germany). Genomic DNA was precipitated by the addition of 0.7 vol isopropanol, and collected by centrifugation at 12,000 × *g* for 10 min at room temperature. The supernatant was removed, and the DNA pellet was washed in 70 % ethanol, re-dissolved in TE buffer (10 mM Tris-HCl, 1 mM EDTA pH 7.5) and stored at -20 °C until required.

### Genome sequencing and assembly

The complete genome sequence of SA11 was determined using pyrosequencing of a paired-end 454 GS-FLX sequence library with Titanium chemistry (Macrogen, Korea). Pyrosequencing reads provided 43× coverage of the genome and were assembled using the Newbler assembler version 2.0 (Roche 454 Life Sciences, USA). The assembly process resulted in 39 contigs across 1 scaffold. Gap closure was managed using the Staden package [20] and gaps were closed using additional Sanger sequencing by standard and inverse PCR based techniques.

### Genome annotation

Genome annotation of the SA11 genome was managed as described previously [21]. The genome sequence was prepared for NCBI submission using Sequin [22], and the adenine residue of the start codon of the chromosomal replication initiator protein DnaA (ACH52_0001) gene was chosen as the first base for the genome.

## Genome properties

**Fig. 5:**
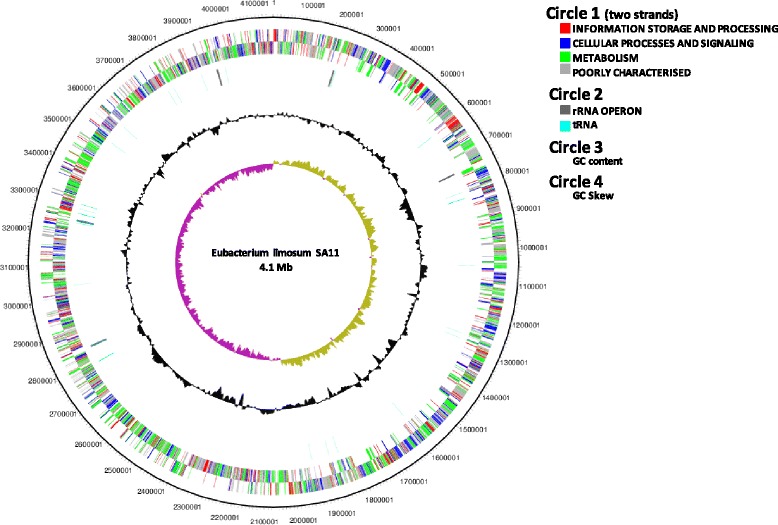
Genome atlas for *E. limosum* SA11. The circles from the outside represent: (1) forward and reverse coding domain sequences (CDS), the colour coding of the CDS represent different Clusters of Orthologous Groups (COG) categories; (2) rRNA and tRNA; (3) % GC plot; (4) GC skew [(GC)/(G + C)]

The genome of *E. limosum* SA11 consists of a single 4,150,332 basepair (bp) circular chromosome with an average G + C content of 47.4 %. A total of 3902 genes were predicted, of which 3805 were protein-coding genes. The properties and statistics of the SA11 genome are summarized in Tables 3 and 4, and the nucleotide sequence has been deposited in Genbank under accession number CP011914. The genome atlas for *E. limosum* SA11 is shown in Fig. 5. Three other *E. limosum* strains have had their genome sequences determined. These are the closed genome of strain KIST612 (4,276,902 bp) isolated from an anaerobic digester [23], the draft genome of the type strain ATCC 8486^T^ (4,370,113 bp) isolated from human faeces [24], and the draft genome of strain 32_A2 isolated from a deep subsurface shale carbon reservoir (Project ID: Gp0114934).

**Table 3:** Genome statistics

Attribute	Value	% of total
Genome size (bp)	4,150,332	100.00
DNA coding (bp)	3,663,440	88.27
DNA G + C (bp)	1,968,558	47.43
DNA scaffolds	1	100.00
Total genes	3902	100.00
Protein coding genes	3805	97.51
RNA genes	76	1.95
Pseudo genes	21	0.54
Genes with function prediction	2856	75.06
Genes assigned to COGs	2545	66.89
Genes with Pfam domains	3349	88.02
Genes with signal peptides	242	6.36
Genes with transmembrane helices	1011	26.57
CRISPR repeats	2

**Table 4:** Number of genes associated with the general COG functional categories

Code	Value	% of total^a^	Description
J	150	3.94	Translation
A	0	0.00	RNA processing and modification
K	314	8.25	Transcription
L	123	3.23	Replication, recombination and repair
B	0	0.00	Chromatin structure and dynamics
D	26	0.68	Cell cycle control, mitosis and meiosis
V	87	2.29	Defense mechanisms
T	168	4.42	Signal transduction mechanisms
M	139	3.65	Cell wall/membrane biogenesis
N	18	0.47	Cell motility
U	15	0.39	Intracellular trafficking and secretion
O	72	1.89	Posttranslational modification, protein turnover, chaperones
C	187	4.91	Energy production and conversion
G	180	4.73	Carbohydrate transport and metabolism
E	272	7.15	Amino acid transport and metabolism
F	65	1.71	Nucleotide transport and metabolism
H	107	2.81	Coenzyme transport and metabolism
I	52	1.37	Lipid transport and metabolism
P	117	3.07	Inorganic ion transport and metabolism
Q	26	0.68	Secondary metabolites biosynthesis, transport and catabolism
R	266	6.99	General function prediction only
S	161	4.23	Function unknown
-	1260	33.11	Not in COGs

^a^The total is based on the total number of protein coding genes in the genome

## Insights from the genome sequence

### Cell envelope

Chemical analysis of the cell wall of the type strain of *E. limosum* (ATCC 8486^T^) shows the presence of the amino sugars *N*-acetylmuramic acid (2.9 % dry weight), *N*-acetylglucosamine (2.1 %) and *N*-acetylgalactosamine (3.9 %) together with larger amounts of rhamnose (20.4 %), glucose and galactose (together 14.9 %). Amino acids identified as present in peptidoglycan were alanine (3.6 %), glutamic acid (8.0 %), lysine (9.0 %), ornithine (12.1 %) and serine (3.4 %) and a putative structure of the peptidoglycan was proposed [25]. In strain SA11 the genes for peptidoglycan biosynthesis are similar to those from other Gram positive bacteria but without the *mre*BCD genes predicted to control cell shape. The SA11 genome contains a large number of genes predicted to be involved in the synthesis of cell wall glycopolymers. These are ordered in six clusters, (ACH52_0663-687 which contains rhamnose biosynthesis genes, ACH52_1029-1040*, ACH52_1350-1371* which contains sialic acid biosynthesis genes, ACH52_1470-1484, ACH52_1620-1630* and ACH52_2094-2105*). Four of these clusters (marked *) are located next to transposase genes. There are also numerous cell surface proteins which contain a variety of domains. SA11 has one cluster of genes (ACH52_2223-2229) predicted to be involved in the biosynthesis and export of a non-ribosomally synthesised peptide of unknown function. The non-ribosomal peptide synthetase gene (ACH52_2225) encodes a 2442 amino acid protein which shows 90 % identity with a similar protein (also 2442 amino acids) from *E. limosum* KIST612. The genomic location of the non-ribosomal peptide synthetase gene differs in the two strains.

### Mobile elements

**Fig. 6:**
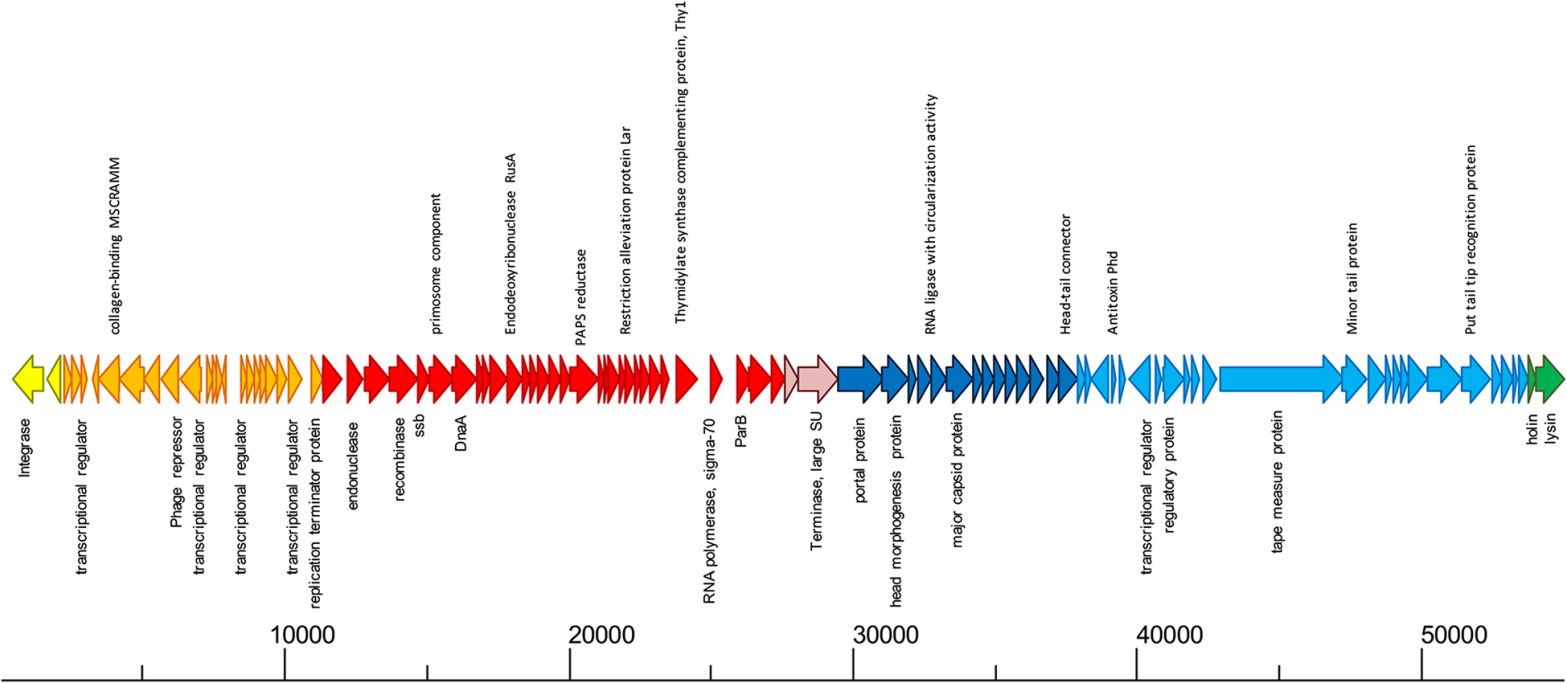
Genome organization of the prophage from *E. limosum* SA11. ORFs are drawn to scale and annotations are shown in vertical text. The absolute size of the phage genome is indicated as a horizontal bar below the genome map, and the numbers indicate nucleotide position

SA11 has a 55 kb prophage (Fig. 6) integrated into the genome (ACH52_1707-1805) adjacent to a serine tRNA. Strain KIST612 does not have a prophage at this location but has three prophages at other sites on the chromosome. In terms of phage defense systems the SA11 chromosome has one cluster of CRISPR genes and two spacer regions at the same locations as found in strain KIST612, but does not contain genes for components of restriction/modification systems. However, there is a gene for a restriction alleviation protein (ACH52_1751) located in the prophage. In addition to the prophage several other gene clusters appear to have been acquired by horizontal transfer. These include all six of the cell wall glycopolymer gene clusters as well as genes for a type VII secretion system (ACH52_0209-0234), cell surface proteins (ACH52_0843-0846), and genes of unknown function ACH52_1057-1076, ACH52_1256-1271, and ACH52_3658-3696). SA11 also has chemotaxis genes (ACH52_0307-0324 and ACH52_3642-3645) which are not present in strain KIST612, but the function of these is unknown as no flagella genes are found in either genome.

### Metabolism

SA11 has a large repertoire of genes involved in central metabolism and grew with hydrogen, formate, some sugars, some compounds containing methoxyl-groups such as methanol and methoxylated benzoates, lactate and pyruvate. These are all typical energy sources for homoacetogenic bacteria.

#### The Wood-Ljungdahl pathway and energy conservation

The Wood-Ljungdahl pathway is central to the metabolism of acetogens and the genes encoding this pathway are found in three distinct clusters in SA11 (ACH52_291-295, ACH52_2912-2912, ACH52_3087-3089) as has been reported for strain KIST612 [26]. SA11 produced only acetate from hydrogen plus carbon dioxide and from glucose, consistent with the use of the Wood-Ljungdahl pathway. Energy conservation in the Wood-Ljungdahl pathway and in acetogens in general has been the focus of extensive study but is not yet fully understood [27]. Key elements of energy conservation systems in *E. limosum* are the membrane-bound Na^+^-translocating Rnf (ACH52_1410-1415) and ATP synthase complexes [26]. As reported for strain KIST612 [26], SA11 has two sets of ATP synthase genes which show different gene orders (ACH52_1610-1617 and ACH52_1920-1928).

#### Polysaccharides

In contrast to most rumen bacteria, SA11 has very few genes encoding glycoside hydrolases. There are two genes encoding GH3 family proteins, one of which (ACH52_0577) has a signal peptide and probably also has a role in cell wall biosynthesis. The gene for a secreted GH4 family protein is located next to an alpha-glucoside specific PTS transport system protein (sa1_0874-0875). SA11 has six genes encoding GH13 family proteins, all of which are predicted to be intracellular and one of which is part of a gene cluster involved in glycogen biosynthesis and degradation (ACH52_0652-0657).

#### Purines

SA11 has a large conserved genetic region associated with selenium-dependent molybdenum hydroxylases (ACH52_1581-1608) [28] which ends with the molybdate ABC transporter genes. The role of these genes in SA11 is not known but it is likely that they encode the selenium-containing xanthine dehydrogenase characterized from the closely related *Eubacterium barkeri* [29].

#### Sugars

Unlike most rumen anaerobes, SA11 has several genes that are either components of, or associated with, PTS carbohydrate transporters [30]. These include PTS transporters for glucose (ACH52_2633) and fructose (ACH52_0805-807) (Fig. 3), as well as glucitol/sorbitol (ACH52_0168-0172, ACH52_1560-1563) and galactitol (ACH52_0007-0009, ACH52_2185-2191).

#### 1,2 propanediol

Rhamnose and fucose are common components of plant cell walls and bacterial exopolysaccharides, and their degradation in the rumen results in lactaldehyde, which is reduced by lactaldehyde reductase to 1,2 propanediol (1,2-PD). There is no literature on the metabolism of 1,2-PD by *E. limosum*, but the acetogen *Acetobacterium woodii* can grow on 1,2-PD producing propionate and propanol as end products [31]. This process occurs independently of acetogenesis. The 1,2-PD degradative pathway has been determined in *Salmonella enterica* and, because the propionaldehyde intermediate is highly toxic to the cell, the process occurs within an organelle called a bacterial microcompartment (BMC) [32]. The BMC consists of a thin protein shell made up of several thousand copies of polypeptides with conserved domains described by the Pfams PF00936 (found in 7 proteins in SA11) and PF03319 (1 protein in SA11). SA11 has a cluster of 19 *pdu* genes encoding degradative enzymes and BMC production (ACH52_0472-490). The gene arrangement is identical to *A. woodii* [31], except the pduO’ gene (Awo_c25780) is not present.

#### Methyl-containing compounds

Pectins make up a significant proportion of plant cell walls and their complex structures are often highly methylated so that action of the enzyme pectin methyl esterase produces methanol in the rumen [33]. *E. limosum* grows well on methanol [15] and has a methanol:corrinoid methyltransferase (ACH52_2073) as part of a larger gene cluster. Phenyl methyl ethers are degradation products of lignin, and their methyl groups can be utilized as carbon and energy sources by acetogens including *E. limosum* [16] and the closely related *E. callanderi* [34]. The ether cleavage is mediated by the *O*-demethylases, which consist of four different proteins: two methyltransferases, a corrinoid protein, and an activating enzyme. SA11 has several genes similar to those described from other bacteria [35] and one gene cluster (ACH52_0344-0347), which is not present in the KIST612 strain, may be involved in the metabolism of these compounds. An unusual feature of the SA11 genome is the presence of multiple copies of genes encoding trimethylamine methyltransferase family proteins (COG05598). SA11 has 39 genes in this category, more than any other bacterial genome, and this seems to be a characteristic of the species as the KIST612 strain has 31 examples. These genes are restricted to the two-thirds of the genome closest to the origin of replication with none found between ACH52_1572 and ACH52_2975. All of these genes are similar in size and predicted to encode proteins between 458 and 492 amino acids. They are usually associated with genes for cobalamin B12-binding proteins (COG05012), BCCT (betaine/carnitine/choline transporter, COG01292, [36]) and MFS family transporters and GntR family transcriptional regulators. Their substrate is not known. *E. limosum* is known to have the ability to demethylate, and thereby increase the bioactivity of, a range of plant isoflavonoids [37–39]. This has led to it being linked with possible health benefits and longevity [40].

### Lactate

Lactate is used for growth by *E. limosum* SA11, and the mechanism of lactate utilization in acetogens has recently been determined in *Acetobacterium woodii* [41]. In this species a stable complex is formed between lactate dehydrogenase and the two subunits of an electron-transferring flavoprotein. This complex uses flavin-based electron bifurcation for energetic coupling. The genes for this complex have been identified in *A. woodii*, and a similar gene cluster is found in *E. limosum* KIST612 [41] and also in SA11 (ACH52_2109-2113).

#### Butyrate

Butyrate is produced when *E. limosum* is grown on a range of substrates and butyrate production by strain KIST612 grown on CO has been studied [26]. The genes for the pathway from acetyl-CoA to butyryl-CoA have been identified and are also found in SA11 (ACH52_3484-3489). The cluster of butyrate genes also includes the two subunits of an electron-transferring flavoprotein (EtfAB) and it is proposed that butyryl-CoA dehydrogenase forms a complex with EtfAB and also uses flavin-based electron bifurcation as reported in *Clostridium kluyveri* [42]. *E. limosum* does not have a butyrate kinase and uses the alternative pathway that transfers the CoA moiety from butyryl-CoA onto acetate (butyryl-CoA:acetate CoA-transferase, ACH52_2647) as the final step in butyrate formation. The SA11 genome contains three EtfAB pairs (ACH52_238-239, ACH52_3175-3176 and ACH52_3178-3179) additional to the ones involved in lactate and butyrate metabolism but the function of these is not known, and is not apparent from their genome context.

## Conclusion

The genome sequence of *Eubacterium limosum* SA11 provides insights into the metabolism of this versatile rumen acetogen. SA11 can grow autotrophically using CO_2_/H_2_ or heterotrophically using a diverse range of substrates with the best growth on glucose or methanol. If autotrophic growth could be encouraged, and hydrogenotrphic methanogens inhibited, then SA11 could be a useful addition to methane mitigation strategies. However, it is apparent that in the rumen SA11 would have a number of different substrates to select from and that autotrophic growth is unlikely to be the norm. Consequently, it is unlikely to be a suitable candidate to take the place of hydrogenotrophic methanogens in the rumen. SA11 does grow well on methanol and it would be interesting to determine if it is able to compete with the methylotrophic methanogens such as *Methanosphaera* species and members of the order *Methanomassiliicoccales* that are present in the rumen.

## Additional file

Additional file 1: Table S1. Associated MIGS record for *M. millerae* SM9, which links to the SIGS supplementary content website. (DOC 70 kb)

## Abbreviations

CH_4_: methane
CO: carbon monoxide
CO_2_: carbon dioxide
H_2_: hydrogen
