# Supplementary material for: The complete genome sequence of Eubacterium limosum SA11, a metabolically versatile rumen acetogen
Source: Stand Genomic Sci. 2016 Mar 15;11:26. doi: 10.1186/s40793-016-0147-9 (PMC4791908; doi:10.1186/s40793-016-0147-9)
Supplement: Additional file 1: Table S1. — Associated MIGS record for M. millerae SM9, which links to the SIGS supplementary content website. (DOC 70 kb) [file 40793_2016_147_MOESM1_ESM.doc]

**Additional file 1**

**Table S1. Associated MIGS record**

| **MIGS-ID** | field name | description |
| --- | --- | --- |
| **MIGS-1** | Submit to INSDC/Trace archives |  |
| **1.1** | PID |  |
| **1.2** | Trace Archive |  |
| **MIGS-2** | MIGS CHECK LIST TYPE |  |
| **MIGS-3** | Project Name | [*Eubacterium limosum*](http://dx.doi.org/10.1601/nm.4229) SA11 |
| **MIGS-4** | Geographic Location | Palmerston North, New Zealand |
| **4.1** | Latitude | -40.35 (40°21'00"S) |
| **4.2** | Longitude | +175.61 (175°36'36"E) |
| **4.3** | Depth | NA |
| **4.4** | Altitude | 30m |
| **MIGS-5** | Time of Sample collection | Not reported |
| **MIGS-6** | Habitat (EnvO) | Sheep rumen |
| **6.1** | temperature | 37^o^C |
| **6.2** | pH | 7.0 |
| **6.3** | salinity |  |
| **6.4** | chlorophyll |  |
| **6.5** | conductivity |  |
|
| **6.6** | light intensity |  |
| **6.7** | dissolved organic carbon (DOC) |  |
| **6.8** | current |  |
| **6.9** | atmospheric data |  |
| **6.10** | density |  |
| **6.11** | alkalinity |  |
| **6.12** | dissolved oxygen |  |
| **6.13** | particulate organic carbon (POC) |  |
| **6.14** | phosphate |  |
| **6.15** | nitrate |  |
| **6.16** | sulfates |  |
| **6.17** | sulfides |  |
| **6.18** | primary production |  |
| **MIGS-7** | Subspecific genetic lineage | Strain SA11 |
| **MIGS-9** | Number of replicons | 1 |
| **MIGS-10** | Extrachromosomal elements | 0 |
| **MIGS-11** | Estimated Size | 4150332 bp |
| **MIGS-12** | Reference for biomaterial or Genome report |  |
| **MIGS-13** | Source material identifiers | Sheep rumen contents |
| **MIGS-14** | Known Pathogenicity | Not known as a pathogen |
|
| **MIGS-15** | Biotic Relationship | Rumen symbiont |
| **MIGS-16** | Specific Host | Sheep |
| **MIGS-17** | Host specificity or range (taxid) | Ruminants |
| **MIGS-18** | Health status of Host | Healthy |
| **MIGS-19** | Trophic Level | Acetogen |
| **MIGS-22** | Relationship to Oxygen | Strict anaerobe |
| **MIGS-23** | Isolation and Growth conditions | Cultured from a sheep rumen sample |
| **MIGS-27** | Nucleic acid preparation |  |
| **MIGS-28** | Library construction | Paired-end library |
| **28.1** | Library size |  |
| **28.2** | Number of reads | 987730 |
| **28.3** | vector | NA |
| **MIGS-29** | Sequencing method | Pyrosequencing |
| **MIGS-30** | Assembly |  |
| **30.1** | Assembly method | Newbler |
| **30.2** | estimated error rate |  |
| **30.3** | method of calculation |  |
| **MIGS-31** | Finishing strategy |  |
| **31.1** | Status | Closed |
| **31.2** | coverage | 43x |
| **31.3** | contigs | 1 |
| **MIGS-32** | Relevant SOPs |  |
| **MIGS-33** | Relevant e-resources |  |
